# Mixed methods in promoting contraceptive decision-making after voluntary-termination-of-pregnancy

**DOI:** 10.3389/fgwh.2025.1543778

**Published:** 2025-02-28

**Authors:** Sara Palma, Ricardo São-João, Mónica Antunes, Helena Presado

**Affiliations:** ^1^ Nursing Research Innovation and Development Centre of Lisbon (CIDNUR), Nursing School of Lisbon (ESEL), Lisbon, Portugal; ^2^ Department of Obstetrics, Santarém Nursing School, Polytechnic Institute of Santarém, Santarém, Portugal; ^3^ Nursing Research Platform Lisbon of the Health Research Center (CIIS), Portuguese Catholic University, Lisbon, Portugal; ^4^ Department of Computer Science and Quantitative Methods, School of Management and Technology, Polytechnic Institute of Santarém, Santarém, Portugal; ^5^ CEAUL—Centro de Estatística e Aplicações, Faculdade de Ciências, Universidade de Lisboa, Lisbon, Portugal; ^6^ Department of Obstetrics, São João de Deus Nursing School, Évora University, Évora, Portugal; ^7^ Department of Obstetrics, Nursing School of Lisbon (ESEL), Lisbon, Portugal

**Keywords:** contraception, contraceptive advice, mixed studies, nurse-midwife, voluntary-termination-of-pregnancy

## Abstract

**Aim:**

Contraception aims to protect women from unplanned and unwanted pregnancies. The number of voluntary-terminations-of-pregnancy is an indicator that highlights issues such as difficulty accessing health services and women's lack of knowledge about contraceptives. These factors complicate choosing, adhering to, continuing, and achieving satisfaction with a contraceptive method. Mixed studies have gained prominence in health research with significant implications for care quality, particularly in nursing.

**Objectives:**

Analyze the applicability of mixed-method research in promoting contraceptive decision-making for women undergoing voluntary termination of pregnancy.

**Methods:**

A theoretical-reflective essay based on a theoretical framework guiding reflections on mixed research. This reflection explores the basic theoretical constructs of mixed methods and their applicability in promoting contraceptive decision-making for women in the process of voluntary pregnancy termination.

**Results:**

Categories emerging from qualitative study participants' statements were consolidated with quantitative data from women's responses to questionnaires. Integrating these two data types facilitated a robust analysis, discussion, and inference of results, leading to proposals for future interventions.

**Conclusions:**

Due to the advantages of the multimethod approach, we aim to disseminate its use in health research, demonstrating that combining quantitative and qualitative approaches provides greater insights into research phenomena and problems compared to using each method in isolation. This ultimately enhances care quality and contributes to scientific knowledge development.

## 1 Introduction

Globally, a significant number of pregnancies are unplanned and result in voluntary-termination-of-pregnancy (VTP). These outcomes are often linked to insufficient health service responses, inadequate contraceptive counseling, and incorrect or discontinuous use of contraceptives (1–3).

The primary goal of contraceptive methods is to protect women, couples, and families from unplanned and unwanted pregnancies (4). In Portugal, access to contraceptives is free through the National Health Service. However, this does not guarantee adherence, correct use, or even adoption of a contraceptive method (1, 5). In some instances, challenges accessing health services and contraceptives persist (2, 6, 7). These challenges were exacerbated during the COVID-19 pandemic, which increased population isolation and led to health care system restructuring to prioritize respiratory infections (8, 9).

In Portugal, women can legally undergo VTP by choice within the first 10 weeks of pregnancy (5). Since its decriminalization in 2007, the number of VTPs decreased until 2011, maintaining a stable trend until 2020 (10, 11). However, a reversal occurred between 2021 and 2022, with a 15% increase in cases (12, 13). This increase may reflect difficulties accessing contraceptives, especially long-term methods, and family planning consultations (8). Lisboa e Vale do Tejo remain the regions with the highest VTP rates nationwide (13).

Despite accessible information on contraceptive methods, unplanned and unwanted pregnancies persist (6). These outcomes are linked to non-use of contraception (14, 15) and a decline in women attending family planning consultations prior to undergoing VTP (13). These trends may indicate service access barriers (8).

Women face significant challenges in making decisions about sexual and reproductive health, particularly in selecting a contraceptive. These challenges are often influenced by cultural beliefs, poverty, limited health literacy, and barriers to accessing health services (5, 16, 17).

After VTP, 93% of women choose to use a contraceptive method, making this period ideal for counseling (13). Health professionals, particularly nurse-midwives, play a key role in this process. Their proximity to women during VTP care and their responsibility for high adherence and continuation of selected methods make them the ideal providers of post-abortion contraceptive counseling (18, 19), should be responsible for providing advice on contraceptive methods after an abortion (20).

Investments in women's health promotion are crucial to empowering women to choose a contraceptive method. Counseling after VTP helps ensure adherence, continuation, and satisfaction with a chosen method as fertility resumes immediately (2, 6, 7, 21, 22).

Based on this problem, a mixed-methods study was carried out to answer the question: “How do obstetric nurses promote the process of deciding on a contraceptive method after voluntary abortion?”, in a health institution in the Lisboa e Vale do Tejo region.

With this theoretical-reflective essay, we intend to analyse the applicability of mixed approaches in research on promoting women's contraceptive choices after voluntary abortion.

## 2 Methods

This theoretical-reflective-essay was grounded in a theoretical framework guiding reflections on mixed-method research. As a reflection article, no inclusion or exclusion criteria were established for material selection. The theoretical constructs used were based on publications about mixed methods, following Creswell (23–25), regardless of temporal limitations, to capture the relevant discourse. Data analysis focused on the ontological, epistemological, and methodological pillars of mixed methods and their benefits in studying quality contraceptive counseling for women post-VTP.

The analysis sought to connect the basic theoretical constructs of mixed methods with their applicability to promoting contraceptive decision-making for women undergoing VTP.

Mixed-method research is widely utilized in health studies because research questions and problems can be comprehensively addressed, enabling better possibilities for analyzing and understanding phenomena (23–27).

The epistemological and ontological challenges of mixed studies complement each other. On one hand, the positivist perspective values neutrality, rationality, and objectivity (28, 29). On the other hand, the interpretative perspective interprets social reality using qualitative data grounded in social sciences. These distinct approaches require different procedures but are mutually reinforcing.

Qualitative research employs data collection techniques such as individual or group interviews, field observations, and historical and ethnographic analyses. This approach provides insights into participants' experiences, behaviors, beliefs, attitudes, and the meanings of studied phenomena. Due to its subjective nature, qualitative research tends to focus on fewer cases (24, 30). Data are generated through direct observation, interaction, and the researcher's interpretation of human experiences, addressing the limitations of quantitative methods (30).

Quantitative data are analyzed using statistical tools, often derived from questionnaires. This approach provides objective insights into large populations, beginning with a theory and following an inductive reasoning process (24, 31, 30). Positivism advocates for observable and empirically testable phenomena using logic and mathematics (29).

Individually, both approaches have limitations and strengths. However, when integrated into a single investigation with distinct purposes, they combine their strengths, yielding comprehensive and consistent research findings (27, 31).

Using a mixed-methodology, we conducted research aimed at answering the question: “How do nurses-midwives promote the decision-making process for contraceptive methods after voluntary termination of pregnancy?” Six objectives were outlined: Investigate nursing practices related to contraceptive counseling in post-VTP consultations; Identify training needs of nurse-midwives in contraception and counseling strategies; Understand the factors identified by nurse-midwives as promoters of contraceptive decision-making; Analyze women's attitudes and knowledge regarding contraception; Evaluate women's perceptions of the contraceptive counseling received from nurse-midwives; Propose nursing interventions in contraceptive counseling to enhance women's decision-making processes.

According to the problem, the research question and the proposed objectives, a study with a multi-method approach was developed, with a concurrent or simultaneous data strategy (23, 27, 32). There are two components of the study: the central component and a secondary component, which, in combination, aim to integrate the information collected and provide a broad perspective on the phenomenon under study (23), thus allowing the design of nurse-midwives interventions that help train women to choose, adhere to and continue using a contraceptive method that meets their needs. It would not be possible to design nurse-midwife interventions tailored to the needs of the women attending the VTP consultation that would respond to the objectives and research question without knowing the nurse-midwives' opinion on the aspects related to the counselling they provide, such as the women's characteristics, attitudes, knowledge and perceptions of the quality of the counselling received.

Following a comprehensive review of the extant literature, it was determined that there was a paucity of knowledge regarding this phenomenon in the Portuguese context. This finding enabled the situation to be diagnosed and the investigation to be justified.

Creswell (23) asserts that multimethod studies comprise two components: one of which can be central, and the other supplementary or identical. The data can be known simultaneously or sequentially. The researcher must decide which study is central, supplementary, or whether they intend to assign the same methodological weight according to the objectives they wish to answer.

In the present theoretical-reflexive-essay, the central component is the qualitative study, the aim of which is to unearth the perceptions, beliefs and feelings of nurse-midwives regarding the contraceptive counselling given to women. The consultation after VTP and the factors that identify as promoters or limitations of women's contraceptive decision-making (5, 28, 33). The study participants comprised nurse-midwives who were part of the consultation at the institution where the study was conducted and who met the previously defined inclusion and exclusion criteria (i.e., they participated in the VTP consultation of the selected hospital unit; they were not study researchers; they were nurse-midwives; they performed contraceptive counselling; and they agreed to participate in the study). The focus group method was employed, as it was considered to be the most effective approach to reach the universe of participants through the debate of ideas, discussions focused on specific themes, which emerge from the interaction between the participants and the researcher (28, 34). A semi-structured interview guide divided into four sections (initial questions, intermediate, final and closing questions), was utilised to explore the topic of contraceptive counselling conducted by nurse-midwives within the framework of the VTP consultation. Additionally, a set of closed questions was employed to characterise the sociodemographic profile of the participants.

The study is underpinned by the analysis of the data collected through the speeches of nurse-midwives, as outlined in the Content Analysis Theory proposed by Bardin (35). The analysis corpus was integrated into the qualitative data analysis software WebQda®, a programme that facilitated enhanced efficiency and rigour in the data analysis process. This approach also enabled collaborative research between researchers, as evidenced by Costa & Amado (36).

In the quantitative analysis (a supplementary component of the study), a questionnaire composed of three scales was applied in order to respond to a set of research questions. This approach enabled the discovery of cause-effect relationships, thus allowing the measurement of attitudes, knowledge and the way in which women evaluate the quality of contraceptive advice given to them. This information was later classified and analysed. The study population comprised women who resorted to VTP consultation. The quantitative approach of the study ensured the representativeness of the results by calculating the sample size. The initial sample size was determined to be 115, considering the potential for incomplete or inaccurate responses. This number was augmented by 30% to account for potential nonresponse and noncompletion, resulting in a final sample size of 172. The study's statistical significance level was set at 5%. The observed response rate was 86% (172/200), indicating a high level of participation.

A consecutive non-probabilistic sample was collected in accordance with the predetermined inclusion and exclusion criteria, which included women aged 16 years or older who were capable of making independent decisions, had undergone VTP, were proficient in Portuguese, could read and write, and had not previously participated in the study. The selection of variables was informed by the extant literature and factors that could influence contraceptive choice. The dependent variable of the study was the women's contraceptive pattern after VTP, while the independent variables included sociodemographic variables, attitudes, knowledge of contraceptives, and the quality of advice received.

The statistical software SPSS (Statistical Package for Social Sciences) version 28 was utilised, employing a range of statistical techniques, including descriptive and inferential statistics.

## 3 Results and discussion

To develop interventions tailored to the needs of both women seeking VTP and the nurse-midwives providing counseling, it is essential to study the perspectives of these stakeholders. In line with the research objectives and questions raised, we determined that a mixed-method approach was necessary. This methodology allows for a comprehensive understanding of the issues by leveraging the strengths of both qualitative and quantitative paradigms.

Following focus group interviews with 5 nurse-midwives and responses to the questionnaire by women attending VTP consultations, results were integrated simultaneously. The five nurse-midwives included in the study were all female, portuguese, with an average age of 47 years, and had been working in VTP/contraceptive counseling for an average of 6.8 years. The nurse-midwives emphasized the importance of establishing a therapeutic and empathetic relationship, which involves listening to women, asking questions, understanding their medical history, expectations, emotions, and contraceptive experiences (5). Barriers highlighted included difficulties in scheduling appointments, insufficient consultation time relative to women's needs, and women's limited knowledge about contraception, influenced by myths, low educational attainment, and a lack of personal accountability. Cultural and linguistic barriers associated with multiculturalism were also noted. Additionally, some nurses reported limited knowledge of contraceptive methods, which hindered the quality of health education provided. They suggested more professional training on counseling strategies, starting in schools, and greater accessibility to services.

The 172 women who responded to the questionnaire were aged between 21 and 30 years and had 12 years of education. Before undergoing VTP, they primarily used oral hormonal contraception or no contraception. Post-counseling, there was an increase in the adoption of long-acting reversible contraceptives (LARCs) and a decrease in non-use of contraception. Women demonstrated low levels of contraceptive knowledge but rated the counseling received from nurses as high quality (37).

The combination of the two studies enabled the formulation of interventions that address emerging needs, as evidenced by the results obtained from interviews conducted with nurse-midwives and questionnaires administered to women attending VTP consultations who provided responses relevant to the research question. Proposed interventions included: a contraceptive nursing consultation and corresponding practice guidelines; a structured contraceptive counseling guide (38); professional training programs on contraception, contraceptive methods, and counseling strategies; online tools to support contraceptive counseling, available in Portuguese and English (39, 40). The development of a curricular unit in a nursing school.

These interventions demonstrated the value of a mixed-method approach in addressing the needs of both women and nurses. Without this methodology, the interventions would have been incomplete, lacking the comprehensive understanding provided by integrating qualitative and quantitative data.

Thus, the validity of quantitative data and the precision of qualitative results were confirmed through rigorous methodology (23).

## 4 Final considerations

Mixed methods combine quantitative and qualitative research techniques, methods, and approaches within a single study. Grounded in pragmatism, they seek practical solutions by analyzing reality.

The concurrent mixed-methodological approach, conducted simultaneously and supported by qualitative and quantitative data collection and analysis techniques, enhanced the results and their interpretation.

Our intention was to synthesize Creswell's methodological assumptions and promote the use of mixed methods in health research. We demonstrated that combining quantitative and qualitative research brings significant advantages, leading to better contraceptive counseling practices and empowering women to choose and use a method effectively.

This study highlights the benefits of mixed-method contributions to scientific knowledge development and evidence-based practice.

## Data Availability

The original contributions presented in the study are included in the article/Supplementary Material, further inquiries can be directed to the corresponding author.
